# Apigenin as a Potential Modulator of Neuroinflammation in Multiple Sclerosis: A Review

**DOI:** 10.3390/biology15171545

**Published:** 2026-09-04

**Authors:** Monique Reis de Santana, Nívia Nonato Silva, Juciele Valéria Ribeiro de Oliveira, Mauricio Moraes Victor, Arthur Morgan Butt, Silvia Lima Costa

**Affiliations:** 1 Laboratory of Neurochemistry and Cellular Biology, Institute of Health Sciences, Federal University of Bahia, Salvador 40231-300, BA, Brazil; monique.reis@ufba.br (M.R.d.S.); nivia.nonato@ufba.br (N.N.S.); juciele.valeria@ufba.br (J.V.R.d.O.); 2 Department of Organic Chemistry, Chemistry Institute, Federal University of Bahia, Salvador 40170-115, BA, Brazil; mmvictor@ufba.br; 3 School of Medicine, Pharmacy and Biomedical Sciences, University of Portsmouth, Portsmouth PO1 2UP, UK

**Keywords:** apigenin, neuroinflammation, multiple sclerosis, experimental autoimmune encephalomyelitis, flavonoid, neuroprotection

## Abstract

Multiple sclerosis (MS) is a chronic inflammatory disease of the central nervous system (CNS) characterized by neuroinflammation and demyelination. Although treatment options for MS have increased over the past decade, current therapies primarily target the peripheral immune system and are often associated with significant side effects. Moreover, these therapies still do not offer solutions for the resulting neurodegeneration that accompanies the progression of the disease. Findings from research using cellular and animal models and studies in humans highlight the neuroprotective and anti-inflammatory properties of apigenin, a flavonoid present in several commonly consumed plant species, such as cabbage, orange, tea, onion, and chamomile. This review compiles chemical and current evidence on the therapeutic potential of apigenin, primarily on its ability to modulate neuroinflammation and underscore the need for further investigation into its clinical applications as an adjuvant therapy for MS.

## Simple Summary

Multiple sclerosis is an autoimmune disease in which the immune system mistakenly attacks the myelin of neurons in the brain and spinal cord, causing chronic inflammation, damage, and disability. Current treatments target the immune system but often bring side effects and fail to stop the ongoing loss of neurons. Naturally occurring compounds are increasingly studied for their ability to modulate neuroinflammation. This review explores apigenin, a natural compound abundantly found in parsley, chamomile, and citrus fruits, focusing on its anti-inflammatory and neuroprotective properties as a potential adjuvant treatment for multiple sclerosis. The available evidence indicates that apigenin lowers overactive immune cell responses, reduces damaging inflammatory signals, and helps to protect neurons. Despite this consistent mechanistic evidence in various animal models, direct data in humans remain limited to a small number of studies on immune cells taken from patients, and no clinical trials have tested apigenin as a treatment. Further research, including clinical trials, is needed to confirm whether apigenin can help to manage and improve treatment of those affected by the disease.

## 1. Introduction

Multiple sclerosis (MS) is a chronic, immune-mediated neurodegenerative disorder of the central nervous system (CNS), characterized by demyelination, axonal damage, and progressive neurological dysfunction [1]. Although its etiology is multifactorial, involving genetic and environmental components, a large body of evidence indicates that neuroinflammation plays a central role in both the initiation and progression of the disease [2]. Importantly, dysregulated neuroinflammatory responses are not exclusive to MS but are also a hallmark of several neurodegenerative disorders, positioning the modulation of neuroinflammation as a key therapeutic target for limiting disease progression and promoting neuroprotection [3].

Neuroinflammation represents a complex and tightly regulated immune response of the CNS, triggered by diverse pathological stimuli such as infection, trauma, ischemia, and toxic insults [4,5]. Under physiological conditions, this response contributes to homeostasis by promoting pathogen clearance, removal of cellular debris, and tissue repair [6]. However, when sustained or dysregulated, neuroinflammation becomes detrimental, and the aberrant activation of peripheral immune cells, along with resident glial cells, leads to excessive production of pro-inflammatory cytokines, chemokines, and reactive oxygen species, ultimately resulting in neuronal dysfunction and irreversible structural damage [7,8].

Glial cells play a pivotal role in orchestrating neuroinflammatory responses. Microglia, the resident immune cells of the CNS, act as first responders to injury, mediating phagocytosis and releasing inflammatory mediators [9]. Astrocytes are essential for maintaining CNS homeostasis, including metabolic support, blood–brain barrier integrity, and neurotransmitter regulation [10]. In response to CNS damage, both cell types undergo phenotypic and functional changes, shifting toward reactive states that amplify inflammatory signaling, promote immune cell recruitment, and contribute to demyelination and neurodegeneration [11].

In this context, naturally occurring bioactive compounds have gained attention as potential modulators of neuroinflammation [12]. Flavonoids, a large class of plant-derived secondary metabolites, are widely distributed in fruits, vegetables, and medicinal plants. Flavonoids exhibit a broad range of biological activities, including antioxidant, anti-inflammatory, and immunomodulatory effects [13]. Among them, apigenin (4′,5,7-trihydroxyflavone), present in food and medicinal plants, has emerged as a promising candidate due to its low toxicity, ability to cross the blood–brain barrier, and capacity to regulate key molecular pathways involved in inflammation and oxidative stress [14]. Accumulating evidence from in vitro and in vivo studies, particularly in models relating to MS and neuroinflammation, highlights the therapeutic potential of apigenin. The effects of apigenin include modulation of glial activation, suppression of pro-inflammatory signaling pathways, and protection against neuronal and myelin damage [15].

In this review, we examine current knowledge on the chemical properties and biological effects of apigenin, with a particular focus on its ability to modulate pathogenic mechanisms of MS. Furthermore, we aim to underscore the need for additional studies to better elucidate apigenin’s mechanisms of action and evaluate its potential as an adjuvant therapeutic strategy for MS.

## 2. Apigenin

### 2.1. Chemical and Biological Aspects

**Figure 1:**
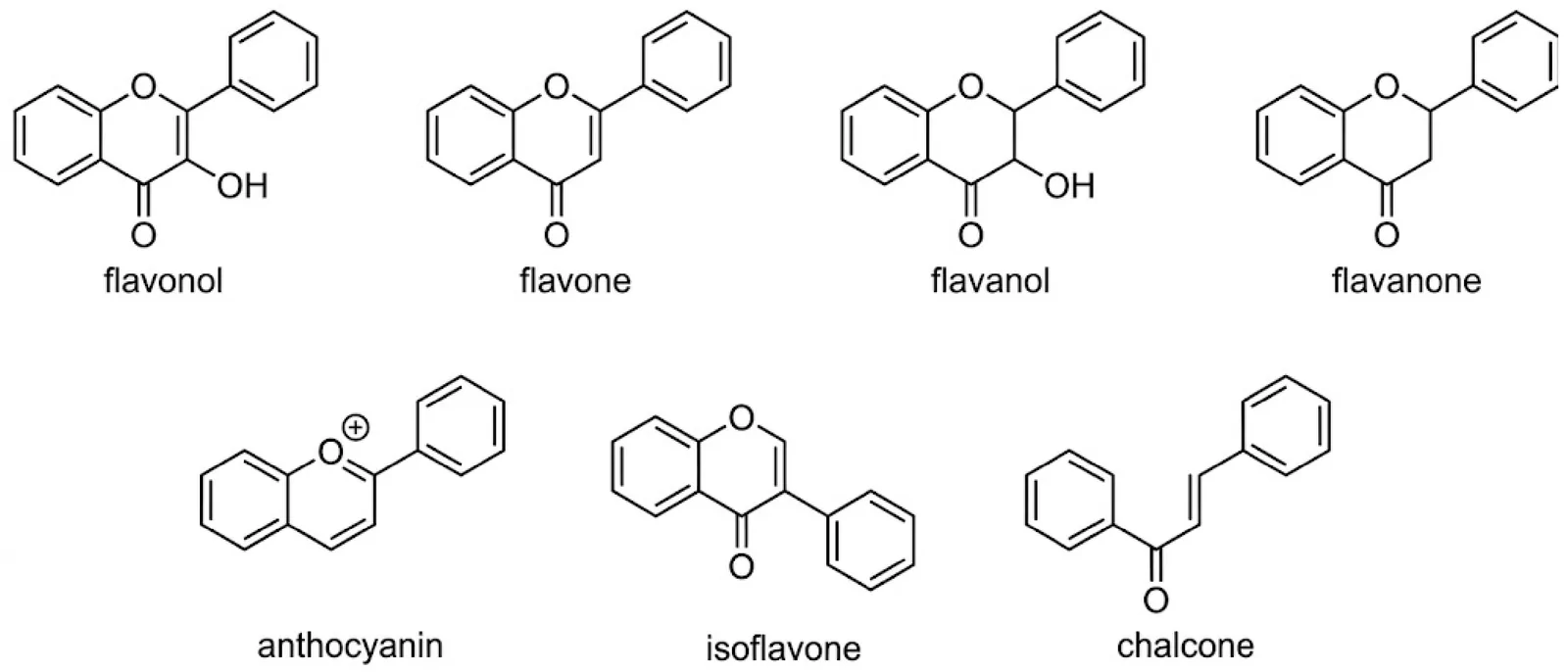
Flavonoid subclasses.

Flavonoids are chemically classified as polyphenols and typically contain one or more hydroxyl groups in their structure. They are divided into subclasses based on their structural differences: flavonols, flavones, flavanones, flavanols, anthocyanins, isoflavones, and chalcones (Figure 1) [16].

**Figure 2:**
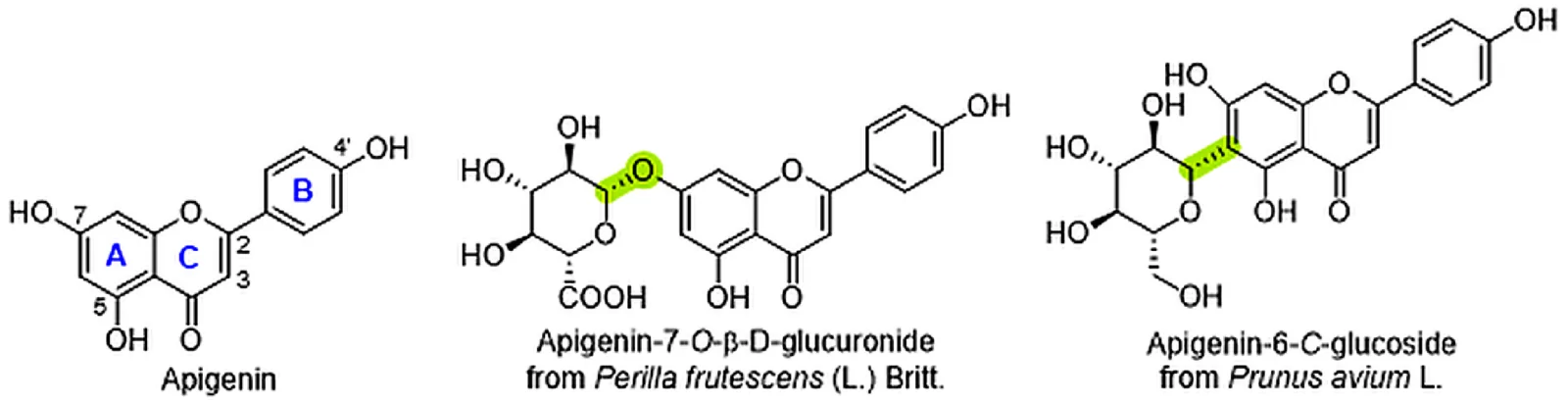
Apigenin and examples of natural O- and C-glycosides. Chemical structures of apigenin and its glycosylated derivatives isolated from *Perilla frutescens* (L.) and *Prunus avium* L. The blue letters (A, B, C) (**left**) denote the standard designation for the structural rings of the flavonoid backbone, and the numbers represent the carbon nomenclature positions. The green shadows highlight the specific glycosidic linkages in each derivative: an O- glycosidic bond at C-7 in apigenin-7-O-β β-D- glucuronoide, and a C-glycosid bond at the C-6 position apigenin-6-C-glucoside.

Apigenin (4′,5,7-trihydroxyflavone) is one of the most common phenolic compounds in the flavone group. It is mainly obtained from the genus Apium (Umbelliferae), being abundant in several fruits, vegetables, and medicinal plants, including oranges, guavas, parsley, celery, onions, oregano, basil, chamomile, and peppermint [17]. The most abundant dietary sources (mg/g) are dried parsley (~45,000), chamomile (3000–5000), and fresh parsley (2100) [18]. Apigenin is structurally composed of a double bond between positions 2 and 3 in ring C, a ketone at position 4 of the C ring, and three hydroxyl groups, with the first and second in the C5 and C7 positions of ring A and the third at C4′ of the B ring [19] (Figure 2). Its molecular formula is C15H10O5, and its molecular weight is 270.24 Da. In nature, it was observed to occur as an aglycone and/or its C- and O-glycosides (detected as 6-C and 8-C-glucoside and 7-O-glucoside), glucuronides, O-methyl ethers, and acetyl derivatives (Figure 2). However, it rarely appears in its free form [15]. Alternatively, apigenin can be easily obtained through chemical reactions from more abundant flavonoids, such as naringin [20].

In recent decades, apigenin has emerged as a promising nutraceutical agent. Several in vitro and in vivo studies have demonstrated its beneficial anti-inflammatory, antioxidant, antiproliferative, pro-apoptotic, and neuroprotective effects [17,21]. Given its therapeutic relevance, the safety and toxicity of this natural compound are critical aspects to consider. Its potential toxicity has been reported. Acute exposure to high doses of apigenin (>100 mg/kg) has been shown to induce hepatotoxicity in Swiss mice related to oxidative stress induction [22]. Furthermore, combined in silico, in vitro, and in vivo evaluations have raised concerns regarding apigenin’s potential for developmental toxicity, endocrine disruption, and mutagenicity [23]. Importantly, these toxic effects were observed at doses substantially higher than those typically employed in therapeutic or experimental models. Experimental studies have indicated that, at low concentrations (0–50 µM), apigenin exhibits low toxicity toward normal cells, suggesting that its safety profile is strongly dose-dependent [24]. In silico toxicity predictions have revealed that apigenin displays both non-carcinogenic and non-mutagenic properties [25]. However, taken together, these studies demonstrate that apigenin’s toxicity must not be overlooked when it is considered for drug development.

### 2.2. Bioavailability (ADME Properties)

The efficacy of a potent natural therapeutic compound depends on its bioavailability, which can be assessed through its absorption, distribution, metabolism, and excretion (ADME) profiles [26]. Numerous studies have investigated the ADME properties of apigenin in both cellular and animal models to support and expand its potential clinical application [27].

Apigenin exhibits limited solubility in aqueous solutions, being partially soluble in water (0.00135 mg/mL) and reaching a solubility of 2.16 μg/mL in phosphate buffer at pH 7.5 [28]. In contrast, apigenin shows high solubility in organic solvents such as dimethylformamide (DMF) and dimethylsulfoxide (DMSO) with reported solubility values of 15 mg/mL and >100 mg/mL, respectively [29]. Therefore, these organic solvents are commonly used to improve apigenin’s solubility in experimental applications. An absorption dynamics study using Caco-2 cells demonstrated that apigenin was progressively absorbed over 240 min, with its uptake peak 30 min after supplementation [30]. Apigenin demonstrates efficient permeability in animal models. In rats, it exhibits concentration-independent permeability, suggesting a predominant passive transport mechanism of absorption across the entire intestine [25]. Moreover, apigenin has shown rapid intestinal uptake and transport compared to other flavonoids such as quercetin [31,32]. Despite these observations, animal studies have reported conflicting results regarding absorption kinetics, with peak plasma levels occurring 3.9 h after administration in some models, while others describe delayed systemic detection up to 24 h post-administration [33,34]. Due to its limited aqueous solubility and high intestinal permeability, apigenin is classified by the Biopharmaceutics Classification System (BCS) as a class II drug [29]. Given that solubility, rather than permeability, is the principal barrier to apigenin’s oral bioavailability, formulation strategies represent the most direct route to enhancing its systemic exposure.

In humans, apigenin absorption has been investigated by monitoring its appearance in plasma and urine over a 24 h period after the ingestion of apigenin-rich food [35]. Studies have reported plasma concentrations of apigenin around 7 h after ingestion (127 ± 81 nmol/L), with only a small fraction of the administered dose recovered in urine [36]. More recent investigations detected urinary excretion of up to 17% of absorbed apigenin, with absence of detectable plasma levels within the first 6 h post-ingestion [35]. Altogether, these findings indicate that, although apigenin shows efficient permeability in experimental models, its absorption kinetics remain incompletely understood and appear to be influenced by experimental conditions and biological context.

Following intestinal uptake, apigenin undergoes significant metabolic transformation involving phase I and phase II enzyme systems [37]. Phase I metabolism, as demonstrated in mouse liver microsomes, is NADPH-dependent and leads to the formation of metabolite luteolin at a measurable rate [38]. Subsequently, phase II reactions are primarily represented by UDP-glucuronosyltransferases (UGTs), resulting in the formation of glucuronide-conjugated metabolites [39]. In parallel, sulfonation, mediated by sulfotransferases (SULTs), also contributes to apigenin metabolism. Notably, in a comparative study evaluating 16 flavonoids, apigenin exhibited the highest sulfonation rates, even at relatively low substrate concentrations (2.5 µM and 10 µM) [40]. Consistent with these findings, studies using murine and human hepatic microsomes have demonstrated rapid and extensive phase II conversion of apigenin [38], while Caco-2 cell monolayer models revealed the generation of a diverse metabolite profile, including multiple conjugated forms distributed across both apical and basolateral compartments [30]. Although this extensive metabolism is often associated with poor bioavailability of flavonoids, it also highlights the formation of a wide range of circulating metabolites that may contribute to the overall biological activity of apigenin, supporting a more integrative perspective of its pharmacokinetic and pharmacodynamic properties.

Finally, distribution is a critical determinant of the biological activity of absorbed compounds. Despite relatively low and delayed plasma concentrations, apigenin has demonstrated the ability to distribute and accumulate in multiple tissues, including the CNS. Experimental evidence indicates that apigenin crosses the blood–brain barrier (BBB), with detectable brain concentrations observed as early as 1.5 h after administration in rats [41,42]. In vitro BBB models further confirm its permeability [43]. The combined pharmacokinetic evidence and demonstrated BBB permeability support the potential of apigenin to exert biological effects within the CNS. However, whether it is sufficient to achieve pharmacologically relevant concentrations remains to be established.

## 3. Pathogenic Mechanisms of Multiple Sclerosis and Potential Modulation by Apigenin

MS is a chronic immune-mediated inflammatory and neurodegenerative disorder of the CNS with unknown etiology. MS progression is characterized by alterations in inflammatory signaling and overproduction of pro-inflammatory biomarkers sustained by infiltrating autoreactive immune cells that infiltrate the CNS across the BBB and activate resident astrocytes and microglia. This chronic inflammatory environment results in the immune-mediated destruction of myelin, a defining feature of MS, compromising axonal integrity and neuronal function. Gradually, demyelination with failed or limited remyelination, driven by peripheral immune infiltration and perpetuated by reactive glial phenotypes, sustains neurotoxicity and drives neurodegeneration [44]. Despite significant advances in disease-modifying therapies for MS, current treatments are limited in their ability to prevent disease progression or the neurodegenerative processes [45]. Moreover, the limited availability of effective therapeutic options underscores the need for novel strategies to overcome the inflammatory and neurodegenerative outcomes of the disease.

**Figure 3:**
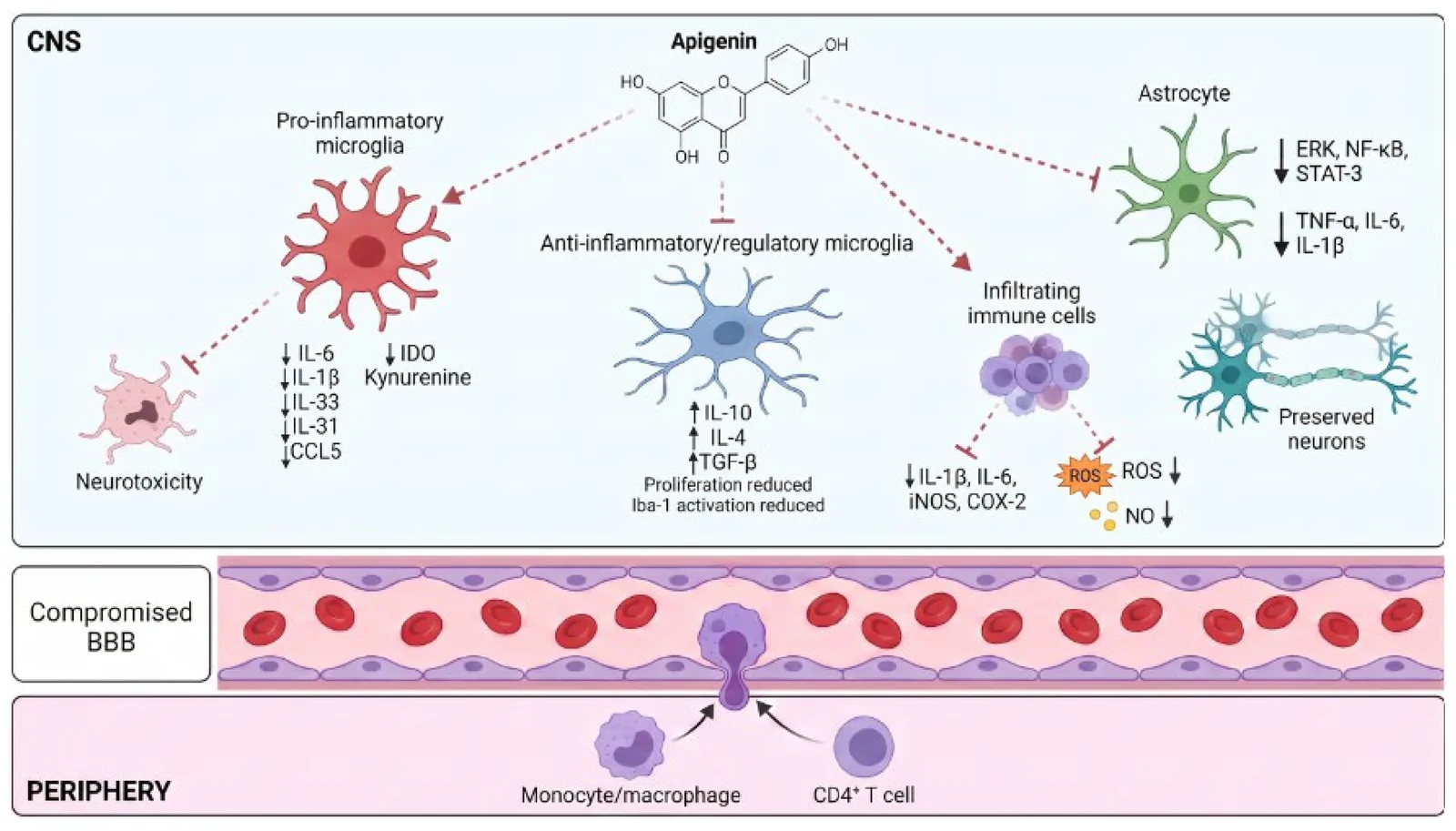
Effects of apigenin reported in different experimental models across immune and CNS-resident cell types, highlighting modulated signaling pathways relevant to MS pathophysiology. Apigenin reduces the production of pro-inflammatory mediators and downregulates inflammatory signaling pathways (downward arrows), while increasing inflammatory and regulatory mediators (upward arrows).

Given that MS pathogenesis involves a complex interplay of chronic inflammation, demyelination, and neurodegeneration, agents capable of simultaneously modulating immune responses and promoting neuroprotection are of particular interest. In this context, apigenin has emerged as a promising candidate in adjuvant therapy for immune-mediated neurodegenerative disorders, due to its reported immunomodulatory and neuroprotective properties across different experimental models, as summarized in Table 1. This section provides an overview of the major pathological mechanisms underlying MS and discusses their potential as therapeutic targets of apigenin. The cellular and molecular effects of apigenin reported across these experimental models are illustrated in Figure 3, highlighting mechanisms that may be relevant to MS pathophysiology.

### 3.1. BBB Disruption

The pathological process of neuroinflammation critically depends on the disruption of BBB integrity, which results in the progression of neurodegenerative diseases [70]. When structurally intact, the BBB plays an important role in the bidirectional exchange of molecules between the blood and brain. The BBB regulates the transport of substances to ensure protection, preventing the passage of toxins and pathogens, and providing optimal function of neurons by regulating the transport of essential molecules and nutrients [71]. The BBB is a complex and dynamic structure composed primarily of endothelial cells interconnected by tight junction complexes including claudin-5 and occludin, and supported by pericytes, astrocytes, microglia cells, and neurons. Together, these cellular components maintain CNS homeostasis as a selectively restricting barrier inhibiting diffusion of macromolecules and immune cells [72,73,74]. In addition to its barrier function, the BBB regulates the influx and efflux of important electrolytes, nutrients, metabolites, and potentially toxic metabolic products through a specialized transport system, thereby maintaining a proper environment required for optimal neuronal activity. It also contributes to immune surveillance by controlling immune cell trafficking and inflammatory molecules [75]. Therefore, any disruption of BBB integrity can impair its protective and regulatory functions and potentially contribute to extensive peripheral immune cell infiltration.

In MS, BBB disruption occurs in the early stages of inflammatory lesion formation, although recurrence can be observed throughout the disease progression [76]. The lesions, on the histopathological level, are characterized by areas of myelin damage with inflammation, axonal degradation, and neuronal loss [77]. During inflammation, BBB disruption is associated with the activation and phenotypic changes in its cellular components and alterations of junctional proteins that compromise barrier integrity and increase permeability. These changes lead to an inflammatory cascade and facilitate recruitment and infiltration of peripheral immune cells into the CNS. Although damage to the BBB is not considered a primary cause of MS or lesion formation, it may contribute to the exacerbation of inflammatory responses of CNS-specific immune cells, thereby promoting neuronal tissue damage [78]. Thus, BBB integrity is a potential therapeutic target to prevent further parenchymal damage.

Evidence of apigenin’s effects on BBB remains limited. Data from a rat model of subarachnoid hemorrhage demonstrated that apigenin reduced neutrophil infiltration and inhibited TLR4-mediated signaling, effects associated with preserved BBB integrity and improved neurological outcome [61]. Furthermore, apigenin modulated the expression of cellular adhesion molecules such as VCAM-1, ICAM-1, and E-selectin in stimulated macrophages [79]. This modulation may contribute to the regulation of peripheral immune cell infiltration across the BBB. However, this evidence derives from a non-MS injury model and a non-endothelial cell type. To our knowledge, no study has directly investigated apigenin’s effect on BBB permeability, junctional protein expression, or adhesion molecule expression directly in an MS-relevant model, highlighting an important gap in the current understanding of its potential effects on BBB dysfunction.

### 3.2. Infiltration and Activation of Peripheral Immune Cells

The mechanisms underlying the pathogenesis of MS are not fully understood. Two complementary paradigms currently frame this discussion: the “outside-in” model, in which a peripherally triggered autoimmune attack against myelin, potentially initiated by molecular mimicry with an infectious agent, is considered the primary event, with CNS damage occurring secondarily; and the “inside-out” model, in which a primary CNS-intrinsic process of oligodendrocyte injury or myelin destabilization releases antigens that secondarily recruit and activate the peripheral immune response [80]. Which of these better describes the earliest pathological changes in MS has not been resolved, and current evidence suggests the two processes may operate concurrently or reinforce one another as the disease progresses. Regardless of which event occurs first, infiltration of peripheral immune cells across a compromised BBB and their local reactivation within the CNS are considered a key driver sustaining MS progression [81].

Among the cell populations involved in this process, cells of the innate immune system, particularly bone marrow-derived myeloid cells, are typically the first to arrive and play a key role in initiating the inflammatory cascade underlying MS onset and progression. Once within the CNS, these infiltrating innate immune cells exert phagocytic and antigen-presenting functions, produce reactive oxygen species (ROS), release pro-inflammatory cytokines and chemokines, and are responsible for reactivating adaptive immune cells (T and B cells), which amplify the immune response of resident glial cells, creating demyelinating lesions that drive MS progression [82]. In experimental autoimmune encephalomyelitis (EAE), an experimental animal model of MS, a number of pro-inflammatory monocytes accumulate rapidly following induction, correlating with the appearance of clinical signs of disease [83,84]. These cells differentiate into macrophages and monocyte-derived dendritic cells (DCs) and are abundantly found in MS active lesions, exerting pro-inflammatory activity, antigen presentation, tissue remodeling, and anti-inflammatory responses [85]. Furthermore, the overexpression of IL12 derived from monocytes and DCs correlates with T lymphocyte differentiation to Th1 and contributes to an autoreactive immune response within active lesions [86]. Given that activation, differentiation, and cytokine signaling in myeloid cells each independently contribute to lesion formation, these cells represent multiple mechanistically distinct targets for therapeutic intervention, several of which, as further discussed, are directly modulated by apigenin. In vitro studies have demonstrated that apigenin exerts immunomodulatory effects on peripheral immune cells. In LPS-induced murine macrophages (RAW264.7), apigenin downregulates the expression of inflammatory mediators such as IL-1β, IL-6, iNOS, COX-2, and NO production [87]. Similarly, in human THP1-derived macrophages, apigenin exhibited its anti-inflammatory effects by suppressing the production of LPS-induced IL-1β, IL6, and TNF-α. Furthermore, it was suggested that apigenin suppresses IL-1β production by inhibiting caspase-1 activation via disruption of NLRP3 inflammasome assembly and reduces IL-6 and IL-1β expression by limiting mRNA stability through ERK1/2 inhibition. Additionally, apigenin attenuates TNF-α– and IL-1β–mediated NF-κB activation [50]. Although most evidence regarding the immunomodulatory properties of apigenin comes from preclinical models (in vitro and in vivo) using human and mouse cells, these findings demonstrate its ability to regulate important pro-inflammatory signaling pathways in peripheral cells, thereby reinforcing its therapeutic potential against neuroinflammation in MS. Consistent with these findings, preclinical studies using the EAE model provide in vivo evidence of the therapeutic potential of apigenin. Directly relevant to myeloid cell functions, apigenin significantly reduces disease severity, progression, and relapse in EAE mice by modulating dendritic cell function: it decreases expression of α4 integrin and CLEC12A on splenic dendritic cells, thereby limiting immune cell infiltration into the CNS [57]. Taken together, these findings position myeloid cells as a mechanistically diverse target of apigenin within MS pathogenesis, though direct evidence from MS patient-derived cells is still lacking. As macrophages and DCs play an important role in activating the T and B lymphocytes, discussed next, their modulation by apigenin may have downstream consequences for the adaptive immune response as well.

Autoreactive CD4+ T cells, particularly the Th1 and Th17 subpopulations, play a central role in the immunopathogenesis of MS [88]. These cells migrate across the BBB and undergo local reactivation within the CNS, where they secrete cytokines and chemokines that initiate and modulate the inflammatory responses associated with MS lesions. Th1 cells produce IFN-γ and TNF-α, cytokines that activate macrophages and promote destruction of myelin-producing oligodendrocytes [89,90]. Th17 cells, in turn, are characterized by the expression of the transcription factor RORγt and production of IL-17A, IL-17F, granulocyte-macrophage colony-stimulating factor (GM-CSF), and other pro-inflammatory cytokines [88]. In particular, the Th17.1 subpopulation, which co-produces IFN-γ and GM-CSF, shows a greater affinity for CNS infiltration and is highly enriched in cerebrospinal fluid from patients with early-stage MS. These cells express high levels of the adhesion molecule VLA-4 (very late antigen-4), facilitating their transmigration across the BBB. Functional deficiency of regulatory T cells (Tregs), which normally suppress Th1 and Th17 lymphocyte activity through IL-10 and TGF-β secretion, represents a central mechanism for perpetuation of the autoimmune response in MS [91]. Given the importance of CD4+ T cells in MS, treatments capable of directly modulating their function or restoring the Treg-mediated regulatory balance are of particular therapeutic interest. As an example, evidence from patient-derived cells supports the immunomodulatory potential of apigenin. It has been demonstrated that apigenin reduces IL-17A expression in Th17 cells derived from peripheral blood mononuclear cells (PBMCs) of MS patients [92]. Additionally, apigenin inhibits Th1 cell proliferation in PBMCs from MS patients in a dose-dependent manner [46]. Complementary evidence from EAE models and human donors shows that apigenin modulates DC-mediated T cell responses, promoting a shift from pro-inflammatory Th1/Th17 phenotypes toward regulatory T cell (Treg) profiles, evidenced by reduced T-bet, IFN-γ, and IL-17, alongside increased IL-10, Transforming Growth Factor-beta (TGF-β), and forkhead box P3 (FoxP3) expression. Mechanistically, these effects are associated with the downregulation of RelB, a critical NF-κB component required for the maturation of DCs and T cell activation, reducing both its expression and nuclear translocation [58]. Overall, these preclinical data position apigenin as a promising multi-target compound capable of modulating key immunopathological processes underlying T cell-driven MS pathogenesis. However, direct evidence in human cells remains limited to a small number of PBMCs-based studies from MS patients, with findings that have yet to be independently replicated. Further studies using larger patient-derived cohorts and complementary MS-related models are needed to validate these observations and determine whether apigenin may have potential as an adjuvant therapeutic strategy for restoring immune balance and limiting neuroinflammation.

B lymphocytes exert fundamental pathogenic roles in MS through both antibody-dependent and antibody-independent mechanisms [93]. B cells function as potent antigen-presenting cells, sustaining encephalitogenic CD4+ T-cell responses via Major histocompatibility complex class II (MHC II)-mediated presentation of myelin and CNS-derived peptides [94]. Autoantibodies against myelin antigens, such as MBP and MOG, actively participate in the demyelination process, being detectable as oligoclonal immunoglobulin G (IgG) bands in cerebrospinal fluid in more than 90% of MS patients [95]. Secretion of IL-6 and TNF-α by B cells promotes differentiation of pathogenic Th17 cells and sustains chronic neuroinflammation in the CNS [96]. Lymphoid follicle-like aggregates containing B cells have been identified in the meninges of MS patients and are associated with more severe neuropathology and accelerated clinical progression, while regulatory B cells (Bregs), producers of IL-10 and TGF-β, are quantitatively and functionally reduced in MS patients, contributing to the immune imbalance that characterizes the disease [93]. No study to date has examined apigenin’s direct effects on B cells in the context of neuroinflammation or MS. In SNF1 mice with an established lupus-like disease model, a distinct antibody-driven autoimmune disease, daily treatment with apigenin (20 mg/kg) inhibited antigen-presenting cells (APCs) function required for Th1 and Th17 cell expansion, and prevented B cells from producing class-switched IgG autoantibodies by up to 82% in vitro, and reduced circulating pathogenic autoantibodies by up to 97% in vivo, delaying the onset of glomerulonephritis. Also, apigenin induced apoptosis of T and B cells by restoring their sensitivity to activation-induced cell death via inhibition of NF-κB-regulated Bcl-xL and c-FLIP [97]. Given the distinct immunopathological nature of MS and lupus, this finding is only suggestive of a possible shared mechanism and represents an evidence gap in apigenin’s modulation of B cells in MS. Further studies using MS patient-derived B cells should investigate whether apigenin modulates antibody production, survival, or antigen-presenting function of B cells.

### 3.3. Glial Cells

#### 3.3.1. Microglia

Beyond peripheral immune cell infiltration contributing to neuroinflammation, CNS-resident cells play a central role in amplifying and sustaining the neuroinflammatory response in MS. Microglial pro-inflammatory responses generate an environment that actively drives neuroinflammation and subsequent neuronal damage, thereby contributing to the irreversible and progressive demyelination process associated with the cognitive decline and poor prognosis of MS [98]. Microglial cells are CNS-resident macrophages that serve as the first line of defense, detecting pathogenic signs through toll-like receptors (TLRs) that sense pathogens or damage-associated molecular patterns (PAMPs and DAMPs), thereby initiating an inflammatory cascade [99].

Microglial activation was historically described using a simplified M1/M2 polarization. This classification remains widely used in the literature but does not adequately reflect the phenotypic and functional heterogeneity of microglia in vivo [100]. The classical pro-inflammatory state, commonly referred to as M1, is generally associated with increased expression of markers such as CD16, CD32, CD86, inducible nitric oxide synthase (iNOS), and MHC-II. In contrast, the alternatively M2 state has traditionally been associated with markers including CD163, CD206, arginase-1, Ym-1, and FIZZ-1 [99]. In reality, microglial responses are considerably more dynamic and context-dependent, encompassing a continuum of transcriptional and functional states rather than discrete polarized phenotypes. Microglia exhibit a phenotypic plasticity that permits them to undertake multiple functional roles in both physiological and pathological contexts [101]. Under physiological conditions, microglia actively survey the surrounding microenvironment [102,103]. The surface molecular repertoire of surveillant microglia encompasses key receptors such as fractalkine receptor (CX3CR1), colony-stimulating factor 1 receptor (CSF1R), integrin CD11b, surface glycoproteins including F4/80, CD68, ionized calcium-binding adaptor molecule 1 (Iba1), and pan-hematopoietic marker CD45, which exhibit lower basal expression levels when compared to circulating macrophage and monocyte populations [104,105].

As discussed previously, infiltration of immune cells into the CNS is associated with inflammatory lesion formation and CNS-resident cell inflammatory response. Under neuroinflammatory conditions, microglia undergo transcriptional and functional changes characterized by their loss of homeostatic gene expression and acquisition of a reactive, pro-inflammatory phenotype. These alterations include enhanced production of pro-inflammatory cytokines, such as interleukins (IL-1 and IL-6), tumor necrosis factor-α (TNF-α), interferon-γ (IFN-γ), increased generation of reactive oxygen species (ROS), and the pathological accumulation of dysfunctional or pH-dysregulated lysosomes, which impair phagocytosis and promote protein aggregation, thereby contributing to neuronal injury and the progression of neurodegenerative disease [106,107]. Given that this “shift” in phenotype is one of the earliest and most consistently observed events in CNS neuroinflammation, understanding whether and how it can be pharmacologically modulated is central to identifying valuable therapeutic strategies for MS.

In this context, apigenin has demonstrated the ability to modulate glial response and suppress cytokine production, including IL-6, TNF-α, and IL-1β in microglial cells, while enhancing release of anti-inflammatory and immune regulatory factors, including IL-4, TGF-β, and IL-10 [108]. Consistently, apigenin inhibits LPS-induced microglial pro-inflammatory response as evidenced by the reduced expression of microglial markers CD11b and CD86 [51]. In mixed neuron–glia coculture systems exposed to inflammatory stimuli, apigenin reduced microglial cell proliferation, modulated Iba-1^+^ cell morphology, and decreased the expression of the inflammatory marker CD68, while shifting cytokine expression toward an anti-inflammatory profile, increasing release of IL-10 and reducing IL-6, IL-1β, and CCL5 [53]. These functional and phenotypic effects on microglia are supported by defined molecular mechanisms. Moreover, it significantly decreased surface CD40 expression, an effect that is suggested to be mediated via the STAT1 signaling pathway [47]. In addition, apigenin decreased LPS-induced TNF-α, IL-6, and IL-1β mRNA and protein levels. Furthermore, microglial activation is suppressed by GSK3β/Nrf2 signaling in a concentration-dependent manner [49]. Beyond classical inflammatory signaling, apigenin’s modulation of microglial response also extends to immunometabolic reprogramming, as seen in its action on the kynurenin pathway. In LPS-stimulated MG6 microglial cells, apigenin suppressed IDO mRNA expression while increasing ACMSD mRNA expression, indicating a potential shift in tryptophan metabolism away from quinolinic acid production. This effect was accompanied by reduced IL-6 and NO production and attenuation of ERK and JNK phosphorylation, together with inhibition of IκBα degradation, suggesting that modulation of tryptophan metabolism may be linked to the anti-inflammatory effects of apigenin through MAPK and NF-κB signaling [25]. Despite the evidence across classical inflammatory and immunometabolic pathways, the translational relevance of these findings to MS remains uncertain. Current evidence derives predominantly from LPS-stimulated microglia cell lines (BV2, MG6, N9, SIM-A9) rather than from MS-relevant models. To date, no study has directly investigated apigenin’s effect on microglia from MS patients or in an MS context. Therefore, studies employing MS-relevant inflammatory stimuli and co-culture models incorporating patient-derived PBMCs with microglia and other MS-inflammatory associated cell types, as well as in vivo models of MS, would further help to determine whether the anti-inflammatory and immunometabolic effects observed are preserved within the complex cellular environment of MS.

#### 3.3.2. Astrocytes

Astrocytes broadly occur as protoplasmic cells in gray matter or fibrous cells in white matter and are important for CNS homeostasis and function [10,109]. These cells perform several important homeostatic functions including the maintenance of fluid and neurotransmitters, induction of synapse formation, and metabolic and neurotrophic support for synapses [110,111]. Additionally, astrocytes participate in neuronal synapse maturation during brain development through the secretion of proteins such as thrombospondins and glypicans that induce synaptogenesis, phagocytic receptors, including MERTK and MEGF10, and regulate the functional integrity of the blood–brain barrier (BBB) [112,113]. Dysregulated or sustained astrocyte reactivity is a central driver of neuroinflammatory cascades that contribute to synaptic dysfunction, neuronal injury, and the progression of diverse neurological disorders [10]. Astrocytes undergo pronounced heterogeneous reactive transformations in response to CNS pathogenic stimuli, spanning inflammatory conditions and neurodegenerative diseases, manifesting as functional, morphological, and molecular reprogramming collectively referred to as astrogliosis [114,115]. Neuroinflammatory microglia drive astrocytes toward a neurotoxic phenotype through secretion of IL-1α, TNFα, and complement component 1q (C1q), cytokines that together are both necessary to induce this neurotoxic astrocyte reactive state, as demonstrated by genetic ablation studies in triple knockout mice [109]. In demyelinating pathology, reactive astrocytes critically impair oligodendrocyte survival and remyelination capacity through astrocytic upregulation of SARM1, which is a central pathological mechanism identified in MS models, such as experimental autoimmune encephalomyelitis (EAE), that suppresses the secretion of glial-derived neurotrophic factor (GDNF) [116]. The sustained activation of Nrf2 in reactive astrocytes represses the cholesterol biosynthesis pathway, as demonstrated by decreased expression of genes such as HMGCS1 and FDPS, thereby preventing the lipid support necessary for oligodendrocytes to survive and effectively remyelinate [117]. Reactive astrocytes can also secrete clusterin (CLU), a glycoprotein highly elevated in active multiple sclerosis lesions that inhibits OPC differentiation directly, induces OPC and oligodendrocyte apoptosis through suppression of PI3K-AKT signaling, and concurrently impairs astrocyte-mediated clearance of myelin debris [118]. Together, these mechanisms establish a sustained pathological state wherein oligodendrocyte regeneration is compromised, resulting in demyelinating lesions.

Apigenin also exerts potent anti-inflammatory effects on astrocytes [119]. In this context, studies in LPS-stimulated astrocytes demonstrate that apigenin inhibits “astrocyte activation”, as evidenced by a reduction in GFAP expression, indicating that astrocytes are a direct cellular target of apigenin. Moreover, apigenin reduces the expression of IL-31 and IL-33 by suppressing key inflammatory signaling pathways, including ERK, NF-κB, and STAT3 [48]. Consistently, in a model of methotrexate-induced hippocampal toxicity, apigenin reduced astrocyte GFAP expression and attenuated neuroinflammation by suppressing TNF-α, IL-6, and NLRP3/IL-1β signaling. These effects were associated with activation of autophagic flux, modulation of the AMPK/mTOR pathway, and downregulation of connexin-43 [120]. Moreover, apigenin significantly suppresses IL-6 expression and regulates astrocytic immune responses through inhibition of CD38, a major NAD^+^-consuming enzyme. These effects were associated with suppression of NF-κB signaling [121]. Importantly, in an in vivo model of fibromyalgia-like pain, apigenin attenuated astrogliosis, as evidenced by reduced GFAP expression, decreased expression of C3, C1q, and S100β, and increased S100A10 levels. Notably, these changes were accompanied by suppression of the kynurenine/aryl hydrocarbon receptor (KYN/AHR) axis, downregulation of IDO and NF-κB signaling, and attenuation of neuroinflammation and oxidative stress, indicating that apigenin modulates astrocyte function through an immunometabolic mechanism [63]. Overall, these findings reinforce the therapeutic relevance of apigenin in targeting astrocyte-mediated inflammatory pathways, while highlighting the need for further studies in MS-relevant models to determine whether these effects translate to the context of MS.

#### 3.3.3. Oligodendrocytes

Accumulating evidence supports oligodendrocyte dysfunction and inflammation-mediated oligodendrocyte apoptosis, substantially important to the pathogenesis and progression of MS [122]. Their loss directly drives chronic demyelination, deprives axons of metabolic and trophic support, rendering axons vulnerable to bioenergetic failure through increased energy demands for saltatory conduction, mitochondrial dysfunction, ionic imbalance, and calcium-mediated degeneration [118]. In response to demyelinating injury, the CNS activates an endogenous repair program, in which oligodendrocyte precursor cells (OPC) proliferate, migrate to lesion sites, and differentiate into newly myelinating oligodendrocytes, partially restoring saltatory conduction and axonal protection [113]. This reparative process is characteristically impaired in chronic MS lesions. Although OPC-mediated remyelination occurs in early relapsing-remitting stages of MS, it is severely restricted in progressive forms of the disease, where OPCs accumulate within lesions but remain blocked from completing their maturation into functional myelinating cells [123,124,125]. Therefore, protecting oligodendrocytes from inflammatory damage or restoring their capacity to complete maturation in this inflammatory environment represents a potential strategy to limit demyelination and subsequent axonal injury in MS.

Evidence suggests that both apigenin and its dimer bis-apigenin (agathisflavone) can exert protective effects on oligodendrocytes. In an ex vivo model of cerebellar slice cultures subjected to oxygen–glucose deprivation, apigenin reduced oligodendrocyte process retraction and myelin loss while increasing neuronal survival, alongside reduced astrocyte reactivity [69]. Previous work from the same research group demonstrated that agathisflavone pretreatment protected acute cerebellar slices against OGD-induced injury [126]. While this provides initial evidence of a direct potential oligodendroglial-protective effect, it derives from an ischemia model rather than an MS-related model and does not address whether apigenin can overcome the specific inhibitory microenvironment, characterized by persistent pro-inflammatory cytokines (TNF-α, IFN-γ, and IL-1β), reactive gliosis, myelin debris, and extracellular matrix remodeling, that blocks OPC maturation in chronic MS lesions. Whether apigenin can restore OPC differentiation under these MS-specific inhibitory conditions remains an open and directly testable question for future research. In this context, the potential of apigenin to preserve oligodendrocyte viability and attenuate demyelination in MS progression remains unexplored, highlighting an important gap for future investigation.

### 3.4. Oxidative and Neuronal Damage

Oxidative stress is implicated in several MS pathological hallmarks, contributing to neuronal damage and disease progression [127]. It arises from disruption of redox homeostasis in which the mitochondrial metabolic generation of ROS exceeds the capacity of cellular antioxidant and repair systems to maintain physiological redox balance, resulting in the accumulation of oxidative damage [128]. In MS, the severity of inflammation is associated with oxidative injury, potentially contributing to axonal damage and neurodegeneration [129].

Studies have demonstrated apigenin’s important antioxidant effects, with the ability to scavenge free radicals. In a zebrafish embryo model, apigenin, along with two other phenolic compounds (rutin and curcumin), protected embryos against oxidative stress-induced lethality as well as induced dysmorphogenesis [130]. Another study showed the significant antioxidant capacity of apigenin by ROS neutralization and ferric reduction through different in vitro assays, including Trolox equivalent antioxidant capacity (TEAC), ferric reducing antioxidant power (FRAP), and oxygen radical absorbance capacity (ORAC) assay [33]. Taken together, these findings indicate that apigenin’s antioxidant activity may represent an additional mechanism underlying its neuroprotective effects in neuroinflammatory conditions. Although direct evidence in MS is lacking, the ability of apigenin to modulate inflammatory responses closely associated with oxidative stress, as discussed above, supports its potential contribution of antioxidant effects to the attenuation of neuroinflammatory and neurodegenerative processes of MS. Apigenin’s antioxidant properties have been associated with delaying the progression of neurodegenerative processes, further supporting its cytoprotective profile [131]. Apigenin also exerts direct neuroprotective actions on neuronal structure and function. Under inflammatory conditions, apigenin preserves neurite integrity and attenuates neuronal apoptosis [132]. Recent evidence from nutritional and systemic models reinforces the role of apigenin as a multi-target immunomodulatory compound capable of regulating key inflammatory and oxidative pathways, particularly NF-κB and Nrf2 signaling. Through these mechanisms, apigenin contributes to the attenuation of neuroinflammatory processes relevant to MS. Notably, ex vivo studies demonstrate that apigenin prevents the loss of myelin sheaths and modulates astrocyte reactivity, as evidenced by increased GFAP-EGFP expression and reduced glutamine synthetase levels [69]. Consistent with these observations, in vivo studies further show that apigenin attenuates LPS-induced neurotoxicity and improves cognitive performance in mice [57]. Collectively, these findings indicate that apigenin preserves neuronal integrity by modulating oxidative stress and apoptotic pathways, highlighting its therapeutic potential in neuroinflammatory and neurodegenerative disorders.

## 4. Conclusions

Taken together, these findings indicate that the flavonoid apigenin may exert neuroprotective effects on several mechanisms relevant to MS pathogenesis, such as BBB integrity, peripheral immune cell infiltration and activation, and CNS-resident cells. By targeting key inflammatory and immune metabolic pathways, apigenin may interfere with both the initiation and progression of neuroinflammation. Across these mechanisms, apigenin’s effects converge repeatedly on NF-κB signaling. Given the central role of dysregulated NF-κB activation in inflammatory diseases, its modulation may represent an important mechanism underlying the anti-inflammatory effects of apigenin, although therapeutic strategies aimed at this pathway must account for the physiological functions of NF-κB and the potential limitations of broad pathway inhibition [132]. From a pharmacodynamic perspective, the available evidence suggests that apigenin acts through multiple molecular targets and interconnected signaling pathways rather than through a single established target. However, most mechanistic evidence derives from non-MS models, and the contribution of these molecular mechanisms to apigenin’s effects in MS remains to be determined.

Although these findings reinforce that targeting neuroinflammation represents a key therapeutic channel in MS, two major limitations restrict the translational interpretation of the available evidence. First, direct evidence in human MS-relevant material is limited, and no clinical trial of apigenin in MS patients has been conducted. Second, apigenin has low aqueous solubility and limited oral bioavailability. The concentrations required to reproduce many of its in vitro effects may exceed what is achievable through dietary intake, and whether comparable effects occur at physiologically attainable exposures is unknown. Thus, these limitations define three priorities for future research: (i) testing apigenin directly in immune cells from MS patients, rather than in non-MS or LPS-induced models; (ii) determining whether its preclinical effects occur at orally achievable concentrations, and, if not, evaluating bioavailability-enhancing delivery strategies; and (iii) clarifying the extent to which NF-κB modulation contributes to apigenin’s effects and whether this occurs through selective modulation of pathological signaling rather than broad pathway inhibition. Apigenin remains a candidate for adjuvant MS therapy on the basis of its favorable safety profile and broad mechanistic activity, but establishing its clinical relevance requires translational gaps to be addressed directly.

## Figures and Tables

**Table 1:** Immunomodulatory and neuroprotective effects of flavonoid apigenin.

Study Design	Experimental Model	Context	Apigenin Concentration	Exposure	Immunomodulatory Effects	Functional Effects	Molecular Targets	Level of Evidence	Year	Ref.
In vitro	Human PBMCs Th1 (CD4^+^/CXCR3^+^)	Multiple Sclerosis	80 µM	48 h	↓ Th1 prolif.; ↓ pro-inflam. genes	↓ Proliferative activity of Th1 (CD4^+^CXCR3^+^)	T-bet (TBX21); IFN-γ	L1	2023	[46]
	Microglia (N9/primary)	IFN-gamma/CD40	25 µM	8 h (FACS/CD40); 12 h(TNF-α/IL-6); 30 min (STAT1)	↓ IFN-γ; ↓ TNF-α; ↓ IL-6	Suppression of microglial activation	STAT1; CD40	L5; L6	2008	[47]
	Astrocytes	LPS-induced	30 and 60 µM	1 h pre-treatment	↓ GFAP; ↓ IL-31 and IL-33	Neuroprotection via microglia inhibition	NF-κB; MAPK (ERK); STAT3	L6	2020	[48]
	Microglia (MG6)	LPS-induced	5, 10, 30, and 50 μM	30 min to 48 h	↑ ACMSD; ↓ IDO; ↓ IL-6, ↓ NO	↓ pro-inflammatory mediators	Kynurenin Pathway (KY); JNK MAPK; NF-κB;	L6	2023	[25]
	Microglia (BV2)	LPS-induced	10, 20 and 40 µM	1 h pre-treatment	↓ TNF-α; ↓ IL-1β; ↓ IL-6	Neuroprotection	GSK3β, Nrf2, HO-1; ↓ NF-κB	L6	2020	[49]
	Human PBMCs (MDMs); Murine BMDMs	LPS-induced	0, 10, 50, and 100 µM in the dose–response	30 min pre-treatment	↓ IL-1β; ↓ Ca2+ flux	Absence of cytotoxicity and preservation of NAD+	NLRP3; CD38; NF-κB/TNF	L5	2025	[50]
	Macrophages (J774A.1; THP1)	LPS-induced	6.25, 12.5, and 25 µM	2 h pre-treatment	↓ IL-1β, ↓ IL-6, ↓ TNF-α; ↑ IL-10; ↓CCL-5; ↓ICAM-1 and VCAM-1	↓ macrophage activation/maturation	NLRP3 (ASC, caspase-1); NF-κB; ERK1/2; cytokine mRNA stability (IL-6 and IL-1β)	L6	2014	[51]
	Macrophages (RAW-264.7)	LPS-induced	0.1, 1, 10, 20, and 30 µM	30 min before or 30 min after LPS	↓ TNF-α; ↓ IL-1β; ↓ IL-10	Attenuation of inflammatory response	Transcriptional regulation of cytokine	L6	2017	[52]
	Glia and neurons (primary)	LPS, IL-1β or Aβ oligomers	1 µM	24 h	↓ Microglial proliferation (↓ CD68, ↓ BrdU); shift M1 → M2 phenotype; ↓ IL-6; ↓ IL-1β; ↓ OX42; ↓ gp130; prevention of astrogliosis	Neuroprotection via microglia inhibition; preservation of neuronal–glial interactions	NF-κB; ERK1/2 (MAPK); NLRP3 inflammasome/caspase-1; IL-6R–gp130 signaling; BDNF pathway	L5	2020	[53]
	Neuronal cells (PC12)	Inorganic arsenic (iAs)–induced	50 µM	1 h pre-treatment	↓ NF-κB activation; ↓ TNF-α, ↓ IL-1β; ↑ GSH; ↑ GPx; ↑ SOD; ↑ CAT; ↓ MDA; ↓ Bax; ↓ caspase-3; ↑ Bcl-2	Neuroprotection; antioxidant activity	Nrf2–ARE (Keap1/Nrf2); NF-κB p65	L6	2023	[54]
	Human PBMCs; (MDMs); Macrophages (RAW 264.7)	LPS-induced	2.5, 5, 20 and 100 μg/mL	1 h pre-treatment	↓ COX-2 activity (high COX-2/COX-1 selectivity); ↓ PGE_2_ production; ↓ macrophage proliferation; ↓ inflammatory mediator release	Reduced inflammatory cell expansion and prostaglandin-driven inflammatory amplification	COX-2 inhibition; prostaglandin E_2_ pathway; oxidative stress pathways	L6	2018	[55]
In Vivo	Spleen, cervical lymph nodes, peripheral blood, brain (mononuclear cells), and spinal cord (cervical and lumbar).	PLP 139-151 peptide stimulation	40 mg/kg body weight	Administered for 5 consecutive days	Modulation of innate immune signaling	Reduced immune cell infiltration and neuroinflammation; decreased EAE disease severity and relapse	MHC II and CD86; α 4 integrin and CLEC12A; IL17A + and CD25 + /FOXP3 +; IFNγ; CD45 + /CD68	L2	2016	[56]
	Hippocampus (CA1 and CA3 regions) and hippocampal pyramidal neurons	LPS-induced	40 mg/kg body weight	Administered for 7 consecutive days	↑ MFN2; ↑ NAD+/NADH; ↑ SIRT3	Neuroprotection via mitochondrial regulation; attenuates cognitive impairment	PGC-1α; TFAM; MFN2; OPA1; PGC-1α; Parkin; LC3II/I	L3	2022	[57]
	Dendritic cell; CD4^+^ T cells;	Autoimmune neuroinflammation	20 µM	3 h	↓ Th1/Th17 differentiation; ↓ DC maturation; ↑ IL-10, ↑ TGF-β, ↑ FoxP3	Reduced demyelination; decreased EAE disease severity; indirect neuroprotection	NF-κB (RelB); TNF-α	L2	2021	[58]
	CNS (brain)	Aluminum chloride (AlCl3)-induced	50 mg/kg	14 consecutive days	↓ AChE; ↓ NO; ↓ MDA; ↓ H2O2; ↑ GSH; ↑ GST activity; ↑ SOD activity	Attenuated neuroinflammation; mitigated neurobehavioral deficits; protected against Purkinje cell layer loss	Oxidative stress pathway	L3	2025	[59]
	Mouse brain tissue	Chronic mild stress-induced depression (CMS)	25 and 50 mg/kg	21 days	↓ Plasma nitrite levels; ↓ ROS; ↑ GSH; ↓ MDA	HPA axis modulation (antidepressant effects); antioxidant activity	MDA; Catalase; GSH; Plasma nitrite; HPA	L3	2022	[60]
	Cerebral cortex neurons; blood–brain barrier; cortical neurons	Subarachnoid hemorrhage (SAH)–induced	10 and 20 mg/kg	30 min	↓ Neutrophil infiltration (↓ MPO activity); ↓ inflammatory response; inhibition of TLR4-mediated signaling; ↓ MDA; ↑ GSH;↓ caspase-3; ↓ Bax	Neuroprotection; BBB protection; improved neurological outcomes	Caspase-3; Bax; Occludina; Claudina-5; MDA; GSH; GSSG; SOD; MPO	L3	2017	[61]
	Hippocampus and cortex neurons)	Acrylonitrile (ACN)–induced	117 mg/kg; 234 mg/kg; 351 mg/kg	30 min	↓ MDA; ↑ GSH; ↑ GSH-Px; ↓ TNF-α; ↓ IL-6; ↓ Cyt-c, ↓ Bax, ↓ caspase-9/3; ↑ Bcl-2/Bax ratio; inhibit neuron apoptosis	Neuroprotection; normalization of brain wet weight and autonomic activity	HMGB1; TLR4; IKK-α; p-IKK-α/β; IκB-α; p-IκB-α; NF-κB p65	L3	2019	[62]
	CNS (spinal cord neurons; astrocytes; microglia)	Reserpine-induced fibromyalgia (FM)	25 mg/kg	14 consecutive days	↓ Astrocyte activation (↓ GFAP); A1 → A2 astrocyte shift (↓ C3, C1q, S100β; ↑ S100A10); ↓ NF-κB, ↓ IL-1β, ↓ IL-18; ↓ IDO/KYN/AHR signaling; ↓ oxidative stress	Neuroprotection; ameliorated spinal cord degeneration	KP–IDO–KYN–AHR axis; NF-κB; oxidative stress pathway; monoaminergic and glutamatergic signaling (NMDA-related)	L3	2026	[63]
	Zebrafish brain	Aluminum-Induced	12 mg/kg; 25 mg/kg; 50 mg/kg	30 consecutive days	↓ SOD, CAT, GSH, GTS, Gpx activity; normalized TBAS and AChE; ↓ APPb, Tau, and inflammatory markers (IL-1β, TNF-α).	Neuroprotection; reduced anxiety- and depression-like behaviors; improved learning and memory; restored social behavior and locomotion	BDNF; mTOR; Appb; Tau	L3	2024	[64]
	Hippocampus; Microglia	IL-6	110 mg/kg	18 months	↓ Microglial activation (↓ Iba-1^+^ microglia); normalization of microglial morphology;	Reduced inflammatory histological markers	IL-6–mediated immune activation	L3	2021	[65]
	Hippocampus neurons	Isoflurane-induced	25 mg/kg; 50 mg/kg; 100 mg/kg	7 days	↓ IL -2; ↓ IL -4; ↓ IL-10; ↓ NF-κB activation; ↑ histone acetylation (H3, H4); ↑ BDNF	Restored cognitive functions; suppressed induced neuroinflammation	Histone acetylation HDAC2 ↓/CBP ↑ balance; BDNF signaling pathway (CAMKII–CREB–ERK); NF-κB (p-p65 and p-IκBα)	L3	2017	[66]
	Hippocampus	Intracerebroventricular STZ-induced depressive-like behavior	10 mg/kg; 20 mg/kg; 40 mg/kg	Single dose 24 h after STZ	↓ ROS; ↓ MDA; ↑ GSH; ↑ CoQ10 ↓ Nlrp3; ↓ Tlr4; ↓ AMPK;	Reduced depressive-like behavior (↓ immobility in FST; ↑ grooming); restored antioxidant capacity and energy homeostasis; regulated inflammatory signaling	Oxidative stress pathways; NLRP3; AMPK	L3	2022	[67]
	Hippocampus	Diet-induced neuroinflammation	1.5 mg/kg	30 days	↓ plasma levels of the pro-inflammatory cytokines (TNF-α, IL-6); ↓ ROS; ↑ SOD, ↑ CAT, ↑ GPx, ↑ GSH	Neuroprotection; preservation of CA1 pyramidal neurons; reduced neuronal loss and shrinkage; improved hippocampal integrity; reduced inflammatory response	3-NT, 4-HNE, p-IKKβ, NF-κB p65, pJNK	L3	2017	[68]
	Oligodendrocytes (SOX10^+^), astrocytes (GFAP^+^), Purkinje neurons	Ischemia (oxygen and glucose deprivation)	10 µM and 20 µM	1 h pre-treatment	↑ GFAP-EGFP; Prevented the reduction of GS	↓ Oligodendrocyte process retraction; ↓ myelin loss; ↑ neuronal survival	MBP; NF70; calbindin D-28K	L4	2026	[69]

Note: ↑ upregulation; ↓ downregulation.

## Data Availability

Data are contained within the article.

## References

[1] Filippi M., Bar-Or A., Piehl F., Preziosa P., Solari A., Vukusic S., Rocca M.A. (2018). Multiple sclerosis. Nat. Rev. Dis. Primers.

[2] Boutitah-Benyaich I., Eixarch H., Villacieros-Álvarez J., Hervera A., Cobo-Calvo Á., Montalban X., Espejo C. (2025). Multiple sclerosis: Molecular pathogenesis and therapeutic intervention. Signal Transduct. Target. Ther..

[3] Shi F.D., Yong V.W. (2025). Neuroinflammation across neurological diseases. Science.

[4] Adamu A., Li S., Gao F., Xue G. (2024). The role of neuroinflammation in neurodegenerative diseases: Current understanding and future therapeutic targets. Front. Aging Neurosci..

[5] Leng F., Edison P. (2021). Neuroinflammation and microglial activation in Alzheimer disease: Where do we go from here?. Nat. Rev. Neurol..

[6] Kim M.E., Lee J.S. (2024). Mechanisms and emerging regulators of neuroinflammation: Exploring new therapeutic strategies for neurological disorders. Curr. Issues Mol. Biol..

[7] Zhang W., Xiao D., Mao Q., Xia H. (2023). Role of neuroinflammation in neurodegeneration development. Signal Transduct. Target. Ther..

[8] Kölliker-Frers R., Udovin L., Otero-Losada M., Kobiec T., Herrera M.I., Palacios J., Capani F. (2021). Neuroinflammation: An integrating overview of reactive-neuroimmune cell interactions in health and disease. Mediat. Inflamm..

[9] Ajoolabady A., Kim B., Abdulkhaliq A.A., Ren J., Bahijri S., Tuomilehto J., Borai A., Khan J., Pratico D. (2025). Dual role of microglia in neuroinflammation and neurodegenerative diseases. Neurobiol. Dis..

[10] Lee H.G., Lee J.H., Flausino L.E., Quintana F.J. (2023). Neuroinflammation: An astrocyte perspective. Sci. Transl. Med..

[11] Bhusal A., Afridi R., Lee W.H., Suk K. (2023). Bidirectional Communication Between Microglia and Astrocytes in Neuroinflammation. Curr. Neuropharmacol..

[12] Hamsalakshmi Alex A.M., Arehally Marappa M., Joghee S., Chidambaram S.B. (2022). Therapeutic benefits of flavonoids against neuroinflammation: A systematic review. Inflammopharmacology.

[13] Li J., Zhao R., Miao P., Xu F., Chen J., Jiang X., Hui Z., Wang L., Bai R. (2023). Discovery of anti-inflammatory natural flavonoids: Diverse scaffolds and promising leads for drug discovery. Eur. J. Med. Chem..

[14] Abid R., Ghazanfar S., Farid A., Sulaman S.M., Idrees M., Amen R.A., Muzammal M., Shahzad M.K., Mohamed M.O., Khaled A.A. (2022). Pharmacological Properties of 4′, 5, 7-Trihydroxyflavone (Apigenin) and Its Impact on Cell Signaling Pathways. Molecules.

[15] Charrière K., Schneider V., Perrignon-Sommet M., Lizard G., Benani A., Jacquin-Piques A., Vejux A. (2024). Exploring the Role of Apigenin in Neuroinflammation: Insights and Implications. Int. J. Mol. Sci..

[16] Yahfoufi N., Alsadi N., Jambi M., Matar C. (2018). The immunomodulatory and anti-inflammatory role of polyphenols. Nutrients.

[17] Salehi B., Venditti A., Sharifi-Rad M., Kręgiel D., Sharifi-Rad J., Durazzo A., Lucarini M., Santini A., Souto E.B., Novellino E. (2019). The Therapeutic Potential of Apigenin. Int. J. Mol. Sci..

[18] Siddiquee R., Mahmood T., Ansari V.A., Ahsan F., Bano S., Ahmad S. (2025). Apigenin unveiled: An encyclopedic review of its preclinical and clinical insights. Discov. Plants.

[19] Calis Z., Mogulkoc R., Baltaci A.K. (2020). The Roles of Flavonols/Flavonoids in Neurodegeneration and Neuroinflammation. Mini Rev. Med. Chem..

[20] Victor M.M., de Carvalho B.C., Dos Santos C.C., Souza C.S., Costa S.L. (2018). Conversion of flavanones to flavones: Synthesis of apigenin from naringenin. Green Process. Synth..

[21] Zare M.F., Rakhshan K., Aboutaleb N., Nikbakht F., Naderi N., Bakhshesh M., Azizi Y. (2019). Apigenin attenuates doxorubicin induced cardiotoxicity via reducing oxidative stress and apoptosis in male rats. Life Sci..

[22] Kumar V., Kundu S. (2022). Studies on Apigenin and Its Biological and Pharmacological Activity in Brain Disorders. Adv. Pharm. Bull..

[23] Singh V.K., Sahoo D., Agrahari K., Khan A., Mukhopadhyay P., Chanda D., Yadav N.P. (2023). Anti-inflammatory, anti-proliferative and anti-psoriatic potential of apigenin in RAW 264.7 cells, HaCaT cells and psoriasis like dermatitis in BALB/c mice. Life Sci..

[24] Zhang X., Wu C. (2022). In Silico, In Vitro, and In Vivo Evaluation of the Developmental Toxicity, Estrogenic Activity, and Mutagenicity of Four Natural Phenolic Flavonoids at Low Exposure Levels. ACS Omega.

[25] Kurniati D., Hirai S., Egashira Y. (2023). Effect of apigenin on tryptophan metabolic key enzymes expression in lipopolysaccharide-induced microglial cells and its mechanism. Heliyon.

[26] Divya Rajaselvi N., Jida M.D., Nair D.B., Sujith S., Beegum N., Nisha A.R. (2023). Toxicity prediction and analysis of flavonoid apigenin as a histone deacetylase inhibitor: An in-silico approach. In Silico Pharmacol..

[27] David S. (2024). Pharmacokinetics: An Overview of Drug Absorption, Distribution, Metabolism and Excretion. J. Pharmacokinet. Exp. Ther..

[28] Wang M., Firrman J., Liu L., Yam K. (2019). A Review on Flavonoid Apigenin: Dietary Intake, ADME, Antimicrobial Effects, and Interactions with Human Gut Microbiota. BioMed Res. Int..

[29] Zhang J., Liu D., Huang Y., Gao Y., Qian S. (2012). Biopharmaceutics classification and intestinal absorption study of apigenin. Int. J. Pharm..

[30] Tang D., Chen K., Huang L., Li J. (2017). Pharmacokinetic Properties and Drug Interactions of Apigenin, a Natural Flavone. Expert. Opin. Drug Metab. Toxicol..

[31] Hsu S.J., Chung H.C., Chang C.H., Liao Y.C., Liu T.W., Lin S.M., Lee C.K. (2025). Rapid evaluation of apigenin bioavailability and hypouricemic bioactivity by targeted metabolomics study in enterohepatic microenvironment mimetic cell culture model. Food Res. Int..

[32] Perez-Moral N., Needs P.W., Kroon P.A. (2018). Rapid intestinal uptake of apigenin compared with quercetin. J. Funct. Foods.

[33] Sánchez-Marzo N., Pérez-Sánchez A., Ruiz-Torres V., de Olivares-Vicente M., Herranz-López M., Micol V., Barrajón-Catalán E. (2019). Antioxidant and pro-oxidant properties of dietary flavonoid apigenin. Int. J. Mol. Sci..

[34] Chen T., Li L.P., Lu X.Y., Jiang H.D., Zeng S. (2007). Absorption and excretion of luteolin and apigenin in rats after oral administration of Chrysanthemum morifolium extract. J. Agric. Food Chem..

[35] Meyer H., Bolarinwa A., Wolfram G., Linseisen J. (2006). Bioavailability of apigenin from apiin-rich parsley in humans. Ann. Nutr. Metab..

[36] Nielsen S.E., Young J.F., Daneshvar B., Lauridsen S.T., Knuthsen P., Sandström B., Dragsted L.O. (1999). Effect of parsley (Petroselinum crispum) intake on urinary apigenin excretion, blood antioxidant enzymes and biomarkers for oxidative stress in human volunteers. Br. J. Nutr..

[37] Borges G., Fong R.Y., Ensunsa J.L., Kimball J., Medici V., Ottaviani J.I., Crozier A. (2022). Absorption, distribution, metabolism and excretion of apigenin and its glycosides in healthy male adults. Free Radic. Biol. Med..

[38] Cassidy A., Minihane A.M. (2017). The role of metabolism (and the microbiome) in defining the clinical efficacy of dietary flavonoids. Am. J. Clin. Nutr..

[39] Cai H., Boocock D.J., Steward W.P., Gescher A.J. (2007). Tissue distribution in mice and metabolism in murine and human liver of apigenin and tricin, flavones with putative cancer chemopreventive properties. Cancer Chemother. Pharmacol..

[40] Gonzales G.B., Smagghe G., Grootaert C., Zotti M., Raes K., Van Camp J. (2015). Flavonoid interactions during digestion, absorption, distribution and metabolism. Drug Metab. Rev..

[41] Meng S., Wu B., Singh R., Yin T., Morrow J.K., Zhang S., Hu M. (2012). SULT1A3-mediated regiospecific 7-O-sulfation of flavonoids in Caco-2 cells. Mol. Pharm..

[42] Shimazu R., Anada M., Miyaguchi A., Nomi Y., Matsumoto H. (2021). Evaluation of Blood-Brain Barrier Permeability of Polyphenols, Anthocyanins, and Their Metabolites. J. Agric. Food Chem..

[43] Mohammadnezhad P., Valdés A., Cokdinleyen M., Mendiola J.A., Cifuentes A. (2025). Evaluation of the In Vitro Blood-Brain Barrier Transport of Ferula persica L. Bioactive Compounds. Int. J. Mol. Sci..

[44] Garton T., Gadani S.P., Gill A.J., Calabresi P.A. (2024). Neurodegeneration and demyelination in multiple sclerosis. Neuron.

[45] Häusler D., Weber M.S. (2024). Towards Treating Multiple Sclerosis Progression. Pharmaceuticals.

[46] Kasiri N., Ghannadian S.M., Hosseini R., Ahmadi L., Rahmati M., Ashtari F., Pourazar A., Eskandari N. (2023). Comparative Analysis of Apigenin-3 Acetate versus Apigenin and Methyl-Prednisolone in Inhibiting Proliferation and Gene Expression of Th1 Cells in Multiple Sclerosis. Cell J..

[47] Rezai-Zadeh K., Ehrhart J., Bai Y., Sanberg P.R., Bickford P., Tan J., Shytle R.D. (2008). Apigenin and luteolin modulate microglial activation via inhibition of STAT1-induced CD40 expression. J. Neuroinflamm..

[48] Che D.N., Cho B.O., Kim J.S., Shin J.Y., Kang H.J., Jang S.I. (2020). Luteolin and Apigenin Attenuate LPS-Induced Astrocyte Activation and Cytokine Production by Targeting MAPK, STAT3, and NF-κB Signaling Pathways. Inflammation.

[49] Chen P., Huo X., Liu W., Li K., Sun Z., Tian J. (2020). Apigenin exhibits anti-inflammatory effects in LPS-stimulated BV2 microglia through activating GSK3β/Nrf2 signaling pathway. Immunopharmacol. Immunotoxicol..

[50] Lauritzen K.H., Yang K., Frisk M., Louwe M.C., Olsen M.B., Ziegler M., Louch W.E., Halvorsen B., Aukrust P., Yndestad A. (2025). Apigenin inhibits NLRP3 inflammasome activation in monocytes and macrophages independently of CD38. Front. Immunol..

[51] Zhang X., Wang G., Gurley E.C., Zhou H. (2014). Flavonoid apigenin inhibits lipopolysaccharide-induced inflammatory response through multiple mechanisms in macrophages. PLoS ONE.

[52] Palacz-Wrobel M., Borkowska P., Paul-Samojedny M., Kowalczyk M., Fila-Danilow A., Suchanek-Raif R., Kowalski J. (2017). Effect of apigenin, kaempferol and resveratrol on the gene expression and protein secretion of tumor necrosis factor alpha (TNF-α) and interleukin-10 (IL-10) in RAW-264.7 macrophages. Biomed. Pharmacother..

[53] Dourado N.S., Souza C.S., de Almeida M.M.A., Bispo da Silva A., dos Santos B.L., Silva V.D.A., De Assis A.M., da Silva J.S., Souza D.O., Costa M.F.D. (2020). Neuroimmunomodulatory and Neuroprotective Effects of the Flavonoid Apigenin in in vitro Models of Neuroinflammation Associated With Alzheimer’s Disease. Front. Aging Neurosci..

[54] Almeer R., Alyami N.M. (2023). The protective effect of apigenin against inorganic arsenic salt-induced toxicity in PC12 cells. Environ. Sci. Pollut. Res. Int..

[55] Makanjuola S.B.L., Ogundaini A.O., Ajonuma L.C., Dosunmu A. (2018). Apigenin and apigeninidin isolates from the Sorghum bicolor leaf targets inflammation via cyclo-oxygenase-2 and prostaglandin-E2 blockade. Clin. Transplant..

[56] Ginwala R., McTish E., Raman C., Singh N., Nagarkatti M., Nagarkatti P., Sagar D., Jain P., Khan Z.K. (2016). Apigenin, a Natural Flavonoid, Attenuates EAE Severity Through the Modulation of Dendritic Cell and Other Immune Cell Functions. J. Neuroimmune Pharmacol..

[57] Ahmedy O.A., Abdelghany T.M., El-Shamarka M.E.A., Khattab M.A., El-Tanbouly D.M. (2022). Apigenin attenuates LPS-induced neurotoxicity and cognitive impairment in mice via promoting mitochondrial fusion/mitophagy: Role of SIRT3/PINK1/Parkin pathway. Psychopharmacology.

[58] Ginwala R., Bhavsar R., Moore P., Bernui M., Singh N., Bearoff F., Nagarkatti M., Khan Z.K., Jain P. (2021). Apigenin Modulates Dendritic Cell Activities and Curbs Inflammation Via RelB Inhibition in the Context of Neuroinflammatory Diseases. J. Neuroimmune Pharmacol..

[59] Oyagbemi A.A., Femi-Akinlosotu O.M., Obasa A.A., Ojo M.S., Salami A.T., Ajibade T.O., Onukak C.E., Igado O.O., Esan O.O., Oyagbemi T.O. (2025). Apigenin mitigates oxidative stress, neuroinflammation, and cognitive impairment but enhances learning and memory in aluminum chloride-induced neurotoxicity in rats. Alzheimers Dement..

[60] Alghamdi A., Almuqbil M., Alrofaidi M.A., Burzangi A.S., Alshamrani A.A., Alzahrani A.R., Kamal M., Imran M., Alshehri S., Mannasaheb B.A. (2022). Potential Antioxidant Activity of Apigenin in the Obviating Stress-Mediated Depressive Symptoms of Experimental Mice. Molecules.

[61] Han Y., Zhang T., Su J., Zhao Y., Wang C., Li X. (2017). Apigenin attenuates oxidative stress and neuronal apoptosis in early brain injury following subarachnoid hemorrhage. J. Clin. Neurosci..

[62] Zhao F., Dang Y., Zhang R., Jing G., Liang W., Xie L., Li Z. (2019). Apigenin attenuates acrylonitrile-induced neuro-inflammation in rats: Involved of inactivation of the TLR4/NF-κB signaling pathway. Int. Immunopharmacol..

[63] Al-Matarneh T.M., El-Said Y.A.M., Ibrahim W.W., El-Tanbouly D.M. (2026). Targeting kynurenine pathway and A1/A2 astrocytes polarization in experimentally induced fibromyalgia: Modulatory role of apigenin on kynurenine/aryl hydrocarbon receptor signaling. Eur. J. Pharmacol..

[64] Boopathi S., Mendonca E., Gandhi A., Rady A., Darwish N.M., Arokiyaraj S., Kumar T.T.A., Pachaiappan R., Guru A., Arockiaraj J. (2024). Exploring the Combined Effect of Exercise and Apigenin on Aluminium-Induced Neurotoxicity in Zebrafish. Mol. Neurobiol..

[65] Chesworth R., Gamage R., Ullah F., Sonego S., Millington C., Fernandez A., Liang H., Karl T., Münch G., Niedermayer G. (2021). Spatial Memory and Microglia Activation in a Mouse Model of Chronic Neuroinflammation and the Anti-inflammatory Effects of Apigenin. Front. Neurosci..

[66] Chen L., Xie W., Xie W., Zhuang W., Jiang C., Liu N. (2017). Apigenin attenuates isoflurane-induced cognitive dysfunction via epigenetic regulation and neuroinflammation in aged rats. Arch. Gerontol. Geriatr..

[67] Bijani S., Dizaji R., Sharafi A., Hosseini M.J. (2022). Neuroprotective Effect of Apigenin on Depressive-Like Behavior: Mechanistic Approach. Neurochem. Res..

[68] Kalivarathan J., Chandrasekaran S.P., Kalaivanan K., Ramachandran V., Carani Venkatraman A. (2017). Apigenin attenuates hippocampal oxidative events, inflammation and pathological alterations in rats fed high fat, fructose diet. Biomed. Pharmacother..

[69] Carreira R.B., dos Santos C.C., de Oliveira J.V.R., Silva N.N., Silva V.D.A.D., Victor M.M., Butt A.M., Costa S.L. (2026). The Flavonoid Apigenin Modulates Oligodendroglial Plasticity and Has a Neuroprotective Effect in Cerebellar Slice Cultures with Oxygen Glucose Deprivation. Nutrients.

[70] Nair A.L., Groenendijk L., Overdevest R., Fowke T.M., Annida R., Mocellin O., de Vries H.E., Wevers N.R. (2023). Human BBB-on-a-chip reveals barrier disruption, endothelial inflammation, and T cell migration under neuroinflammatory conditions. Front. Mol. Neurosci..

[71] Huang X., Hussain B., Chang J. (2021). Peripheral inflammation and blood-brain barrier disruption: Effects and mechanisms. CNS Neurosci. Ther..

[72] Zhao J., Wang A., Li X. (2026). A systematic review and meta-analysis of the relationship between neuroinflammation and blood-brain barrier based on in vitro models. Biochem. Biophys. Rep..

[73] Candelario-Jalil E., Dijkhuizen R.M., Magnus T. (2022). Neuroinflammation, stroke, blood-brain barrier dysfunction, and imaging modalities. Stroke.

[74] Su Y., Wang X., Yang Y., Chen L., Xia W., Hoi K.K., Li H., Wang Q., Yu G., Chen X. (2023). Astrocyte endfoot formation controls the termination of oligodendrocyte precursor cell perivascular migration during development. Neuron.

[75] Garcia-Gallardo A., Campbell M. (2025). Understanding the Blood-Brain Barrier: From Physiology to Pathology. Adv. Exp. Med. Biol..

[76] Ortiz G.G., Pacheco-Moisés F.P., Macías-Islas M.Á., Flores-Alvarado L.J., Mireles-Ramírez M.A., González-Renovato E.D., Hernández-Navarro V.E., Sánchez-López A.L., Alatorre-Jiménez M.A. (2014). Role of the blood-brain barrier in multiple sclerosis. Arch. Med. Res..

[77] Lassmann H. (2018). Multiple Sclerosis Pathology. Cold Spring Harb. Perspect. Med..

[78] Schreiner T.G., Romanescu C., Popescu B.O. (2022). The Blood–Brain Barrier—A Key Player in Multiple Sclerosis Disease Mechanisms. Biomolecules.

[79] Lee J.H., Zhou H.Y., Cho S.Y., Kim Y.S., Lee Y.S., Jeong C.S. (2007). Anti-inflammatory mechanisms of apigenin: Inhibition of cyclooxygenase-2 expression, adhesion of monocytes to human umbilical vein endothelial cells, and expression of cellular adhesion molecules. Arch. Pharm. Res..

[80] ‘t Hart B.A., Luchicchi A., Schenk G.J., Stys P.K., Geurts J.J.G. (2021). Mechanistic underpinning of an inside-out concept for autoimmunity in multiple sclerosis. Ann. Clin. Transl. Neurol..

[81] Kaufmann M., Evans H., Schaupp A.L., Engler J.B., Kaur G., Willing A., Kursawe N., Schubert C., Attfield K.E., Fugger L. (2021). Identifying CNS-colonizing T cells as potential therapeutic targets to prevent progression of multiple sclerosis. Med.

[82] Parsi S., Zhu C., Motlagh N.J., Kim D., Küllenberg E.G., Kim H.H., Gillani R.L., Chen J.W. (2024). Basic Science of Neuroinflammation and Involvement of the Inflammatory Response in Disorders of the Nervous System. Magn. Reson. Imaging Clin. N. Am..

[83] Mishra M.K., Wang J., Silva C., Mack M., Yong V.W. (2012). Kinetics of proinflammatory monocytes in a model of multiple sclerosis and its perturbation by laquinimod. Am. J. Pathol..

[84] Cugurra A., Mamuladze T., Rustenhoven J., Dykstra T., Beroshvili G., Greenberg Z.J., Baker W., Papadopoulos Z., Drieu A., Blackburn S. (2021). Skull and vertebral bone marrow are myeloid cell reservoirs for the meninges and CNS parenchyma. Science.

[85] Guilliams M., Mildner A., Yona S. (2018). Developmental and Functional Heterogeneity of Monocytes. Immunity.

[86] Makhlouf K., Weiner H.L., Khoury S.J. (2001). Increased percentage of IL-12+ monocytes in the blood correlates with the presence of active MRI lesions in MS. J. Neuroimmunol..

[87] Park C.H., Min S.Y., Yu H.W., Kim K., Kim S., Lee H.J., Kim J.H., Park Y.J. (2020). Effects of Apigenin on RBL-2H3, RAW264.7, and HaCaT Cells: Anti-Allergic, Anti-Inflammatory, and Skin-Protective Activities. Int. J. Mol. Sci..

[88] Ishina I.A., Zakharova M.Y., Kurbatskaia I.N., Mamedov A.E., Belogurov A.A., Gabibov A.G. (2023). MHC class II presentation in autoimmunity. Cells.

[89] El-Taibany A.A., Heydarian P., Porada D.A., Seeds M.C., Atala A. (2025). Multiple sclerosis: Etiology in the context of neurovascular unit and immune system involvement. Front. Immunol..

[90] Szabo S.J., Kim S.T., Costa G.L., Zhang X., Fathman C.G., Glimcher L.H. (2000). A novel transcription factor, T-bet, directs Th1 lineage commitment. Cell.

[91] Van Langelaar J., van der Vuurst de Vries R.M., Janssen M., Wierenga-Wolf A.F., Spilt I.M., Siepman T.A., Dankers W., Verjans G.M.G.M., de Vries H.E., Lubberts E. (2018). T helper 17.1 cells associate with multiple sclerosis disease activity. Brain.

[92] Rahmati M., Ghannadian S.M., Kasiri N., Ahmadi L., Motedayyen H., Shaygannejad V., Pourazar A., Alsahebfosoul F., Ganjalikhani Hakemi M., Eskandari N. (2021). Modulation of Th17 Proliferation and IL-17A Gene Expression by Acetylated Form of Apigenin in Patients with Multiple Sclerosis. Immunol. Investig..

[93] Wang A.A., Luessi F., Neziraj T., Pössnecker E., Zuo M., Engel S., Hanuscheck N., Florescu A., Bugbee E., Ma X.I. (2024). B cell depletion with anti-CD20 promotes neuroprotection in a BAFF-dependent manner in mice and humans. Sci. Transl. Med..

[94] Di Sabatino E., Ferraro D., Gaetani L., Emiliano E., Parnetti L., Di Filippo M. (2025). CSF biomarkers of B-cell activation in multiple sclerosis. J. Neurol..

[95] Piancone F., Saresella M., Marventano I., La Rosa F., Zoppis M., Agostini S., Longhi R., Caputo D., Mendozzi L., Rovaris M. (2016). B lymphocytes in multiple sclerosis: Bregs and BTLA/CD272 expressing-CD19+ lymphocytes modulate disease severity. Sci. Rep..

[96] Gharibi T., Babaloo Z., Hosseini A., Marofi F., Ebrahimi-kalan A., Jahandideh S., Baradaran B. (2020). The role of B cells in the immunopathogenesis of multiple sclerosis. Immunology.

[97] Kang H.K., Ecklund D., Liu M., Datta S.K. (2009). Apigenin, a non-mutagenic dietary flavonoid, suppresses lupus by inhibiting autoantigen presentation for expansion of autoreactive Th1 and Th17 cells. Arthritis Res. Ther..

[98] Nan Y., Ni S., Liu M., Hu K. (2024). The emerging role of microglia in the development and therapy of multiple sclerosis. Int. Immunopharmacol..

[99] Shao F., Wang X., Wu H., Wu Q., Zhang J. (2022). Microglia and neuroinflammation: Crucial pathological mechanisms in traumatic brain injury-induced neurodegeneration. Front. Aging Neurosci..

[100] Guo S., Wang H., Yin Y. (2022). Microglia Polarization From M1 to M2 in Neurodegenerative Diseases. Front. Aging Neurosci..

[101] Paolicelli R.C., Sierra A., Stevens B., Tremblay M.E., Aguzzi A., Ajami B., Amit I., Audinat E., Bechmann I., Bennett M. (2022). Microglia states and nomenclature: A field at its crossroads. Neuron.

[102] Gao C., Jiang J., Tan Y., Chen S. (2023). Microglia in neurodegenerative diseases: Mechanism and potential therapeutic targets. Signal Transduct. Target. Ther..

[103] Hanisch U.K., Kettenmann H. (2007). Microglia: Active sensor and versatile effector cells in the normal and pathologic brain. Nat. Neurosci..

[104] Huang J.M., Zhao N., Hao X.N., Li S.Y., Wei D., Pu N., Peng G.-H., Tao Y. (2024). CX3CL1/CX3CR1 signaling mediated neuroglia activation is implicated in the retinal degeneration: A potential therapeutic target to prevent photoreceptor death. Investig. Ophthalmol. Vis. Sci..

[105] Smith J.A., Das A., Ray S.K., Banik N.L. (2012). Role of pro-inflammatory cytokines released from microglia in neurodegenerative diseases. Brain Res. Bull..

[106] Marschallinger J., Iram T., Zardeneta M., Lee S.E., Lehallier B., Haney M.S., Pluvinage J.V., Mathur V., Hahn O., Morgens D.W. (2020). Lipid-droplet-accumulating microglia represent a dysfunctional and proinflammatory state in the aging brain. Nat. Neurosci..

[107] Liddelow S.A., Guttenplan K.A., Clarke L.E., Bennett F.C., Bohlen C.J., Schirmer L., Bennett M.L., Münch A.E., Chung W.-S., Peterson T.C. (2017). Neurotoxic reactive astrocytes are induced by activated microglia. Nature.

[108] Shu N., Zhang Z., Wang X., Li R., Li W., Liu X., Zhang Q., Jiang Z., Tao L., Zhang L. (2023). Apigenin Alleviates Autoimmune Uveitis by Inhibiting Microglia M1 Pro-Inflammatory Polarization. Investig. Ophthalmol. Vis. Sci..

[109] Sofroniew M.V., Vinters H.V. (2010). Astrocytes: Biology and pathology. Acta Neuropathol..

[110] Pekny M., Pekna M., Messing A., Steinhäuser C., Lee J.M., Parpura V., Hol E.M., Sofroniew M.V., Verkhratsky A. (2016). Astrocytes: A central element in neurological diseases. Acta Neuropathol..

[111] Eroglu C., Barres B.A. (2010). Regulation of synaptic connectivity by glia. Nature.

[112] Chung W.S., Clarke L.E., Wang G.X., Stafford B.K., Sher A., Chakraborty C., Joung J., Foo L.C., Thompson A., Chen C. (2013). Astrocytes mediate synapse elimination through MEGF10 and MERTK pathways. Nature.

[113] Profaci C.P., Munji R.N., Pulido R.S., Daneman R. (2020). The blood-brain barrier in health and disease: Important unanswered questions. J. Exp. Med..

[114] Lee H.G., Wheeler M.A., Quintana F.J. (2022). Function and therapeutic value of astrocytes in neurological diseases. Nat. Rev. Drug Discov..

[115] Escartin C., Galea E., Lakatos A., O’Callaghan J.P., Petzold G.C., Serrano-Pozo A., Steinhäuser C., Volterra A., Carmignoto G., Agarwal A. (2021). Reactive astrocyte nomenclature, definitions, and future directions. Nat. Neurosci..

[116] Jin L., Zhang J., Hua X., Xu X., Li J., Wang J., Wang M., Liu H., Qiu H., Chen M. (2022). Astrocytic SARM1 promotes neuroinflammation and axonal demyelination in experimental autoimmune encephalomyelitis through inhibiting GDNF signaling. Cell Death Dis..

[117] Molina-Gonzalez I., Holloway R.K., Jiwaji Z., Dando O., Kent S.A., Emelianova K., Lloyd A.F., Forbes L.H., Mahmood A., Skripuletz T. (2023). Astrocyte-oligodendrocyte interaction regulates central nervous system regeneration. Nat. Commun..

[118] Klistorner A., Klistorner S., You Y., Graham S.L., Yiannikas C., Parratt J., Barnett M. (2022). Long-term effect of permanent demyelination on axonal survival in multiple sclerosis. Neurol. Neuroimmunol. Neuroinflamm..

[119] Brambilla R. (2019). The contribution of astrocytes to the neuroinflammatory response in multiple sclerosis and experimental autoimmune encephalomyelitis. Acta Neuropathol..

[120] Abd El-Aal S.A., El-Sayyad S.M., El-Gazar A.A., Salaheldin Abdelhamid Ibrahim S., Essa M.A., Abostate H.M., Ragab G.M. (2024). Boswellic acid and apigenin alleviate methotrexate-provoked renal and hippocampal alterations in rats: Targeting autophagy, NOD-2/NF-κB/NLRP3, and connexin-43. Int. Immunopharmacol..

[121] Roboon J., Hattori T., Ishii H., Takarada-Iemata M., Nguyen D.T., Heer C.D., O’Meally D., Brenner C., Yamamoto Y., Okamoto H. (2021). Inhibition of CD38 and supplementation of nicotinamide riboside ameliorate lipopolysaccharide-induced microglial and astrocytic neuroinflammation by increasing NAD. J. Neurochem..

[122] García-Domínguez M. (2025). White Matter in Crisis: Oligodendrocytes and the Pathophysiology of Multiple Sclerosis. Cells.

[123] Lin W., Kemper A., Dupree J.L., Harding H.P., Ron D., Popko B. (2006). Interferon-γ inhibits central nervous system remyelination through a process modulated by endoplasmic reticulum stress. Brain.

[124] Åkesson J., Hojjati S., Hellberg S., Raffetseder J., Khademi M., Rynkowski R., Kockum I., Altafini C., Lubovac-Pilav Z., Mellergård J. (2023). Proteomics reveal biomarkers for diagnosis, disease activity and long-term disability outcomes in multiple sclerosis. Nat. Commun..

[125] Carreira R.B., Dos Santos C.C., de Oliveira J.V.R., da Silva V.D.A., David J.M., Butt A.M., Costa S.L. (2024). Neuroprotective Effect of Flavonoid Agathisflavone in the Ex Vivo Cerebellar Slice Neonatal Ischemia. Molecules.

[126] Ramos-González E.J., Bitzer-Quintero O.K., Ortiz G., Hernández-Cruz J.J., Ramírez-Jirano L.J. (2024). Relationship between inflammation and oxidative stress and its effect on multiple sclerosis. Neurologia.

[127] Jiménez-Jiménez F.J., Alonso-Navarro H., Salgado-Cámara P., García-Martín E., Agúndez J.A.G. (2024). Oxidative Stress Markers in Multiple Sclerosis. Int. J. Mol. Sci..

[128] Pegoretti V., Swanson K.A., Bethea J.R., Probert L., Eisel U.L.M., Fischer R. (2020). Inflammation and Oxidative Stress in Multiple Sclerosis: Consequences for Therapy Development. Oxid. Med. Cell Longev..

[129] Arteaga C., Boix N., Teixido E., Marizande F., Cadena S., Bustillos A. (2021). The Zebrafish Embryo as a Model to Test Protective Effects of Food Antioxidant Compounds. Molecules.

[130] Kim M., Jung J., Jeong N.Y., Chung H.J. (2019). The natural plant flavonoid apigenin is a strong antioxidant that effectively delays peripheral neurodegenerative processes. Anat. Sci. Int..

[131] Balez R., Steiner N., Engel M., Muñoz S.S., Lum J.S., Wu Y., Wang D., Vallotton P., Sachdev P., O’Connor M. (2016). Neuroprotective effects of apigenin against inflammation, neuronal excitability and apoptosis in an induced pluripotent stem cell model of Alzheimer’s disease. Sci. Rep..

[132] Liu T., Zhang L., Joo D., Sun S.C. (2017). NF-κB signaling in inflammation. Signal Transduct. Target. Ther..

